# Bioprocessed Brewers’ Spent Grain Improves Nutritional and Antioxidant Properties of Pasta

**DOI:** 10.3390/antiox10050742

**Published:** 2021-05-07

**Authors:** Rosa Schettino, Michela Verni, Marta Acin-Albiac, Olimpia Vincentini, Annika Krona, Antti Knaapila, Raffaella Di Cagno, Marco Gobbetti, Carlo Giuseppe Rizzello, Rossana Coda

**Affiliations:** 1 Department of Soil, Plant and Food Science, University of Bari Aldo Moro, 70126 Bari, Italy; rosa.schettino@libero.it (R.S.); michela.verni@uniba.it (M.V.); 2 Faculty of Science and Technology, Libera Universitá di Bolzano, 39100 Bolzano, Italy; Marta.AcinAlbiac@natec.unibz.it (M.A.-A.); raffaella.dicagno@unibz.it (R.D.C.); marco.gobbetti@unibz.it (M.G.); 3 Unit of Human Nutrition and Health, Department of Food Safety, Nutrition and Veterinary Public Health, Istituto Superiore di Sanità, 00161 Rome, Italy; olimpia.vincentini@iss.it; 4 RISE Research Institutes of Sweden, Agriculture and Food, Box 5401, 402 29 Gothenburg, Sweden; annika.krona@ri.se; 5 Department of Food and Nutrition, University of Helsinki, 00014 Helsinki, Finland; antti.knaapila@helsinki.fi (A.K.); rossana.coda@helsinki.fi (R.C.); 6 Department of Environmental Biology, “Sapienza” University of Rome, 00185 Rome, Italy; 7 Department of Food and Nutrition, Helsinki Institute of Sustainability, University of Helsinki, 00014 Helsinki, Finland

**Keywords:** Brewers’ spent grain, lactic acid bacteria, enzymes, pasta, antioxidant activity, fiber, nutritional values, sensory analysis

## Abstract

Brewers’ spent grain (BSG), the by-product of brewing, was subjected to a xylanase treatment followed by fermentation with *Lactiplantibacillus plantarum* PU1. Bioprocessed BSG has been used as ingredient to obtain a fortified semolina pasta which can be labeled as “high fiber” and “source of protein” according to the European Community Regulation No. 1924/2006. Compared to native BSG, the use of bioprocessed BSG led to higher protein digestibility and quality indices (essential amino acid index, biological value, protein efficiency ratio, nutritional index), as well as lower predicted glycemic index. Bioprocessing also improved the technological properties of fortified pasta. Indeed, brightfield and confocal laser scanning microscopy revealed the formation of a more homogeneous protein network, resulting from the degradation of the arabinoxylan structure of BSG, and the release of the components entrapped into the cellular compartments. The extensive cell wall disruption contributed to the release of phenols, and conferred enhanced antioxidant activity to the fortified pasta. The persistence of the activity was demonstrated after in vitro-mimicked digestion, evaluating the protective effects of the digested pasta towards induced oxidative stress in Caco-2 cells cultures. The fortified pasta showed a peculiar sensory profile, markedly improved by the pre-treatment, thus confirming the great potential of bioprocessed BSG as health-promoting food ingredient.

## 1. Introduction

Brewers’ spent grain (BSG), the main by-product of the beer industry, represents 85% of the total residues from the brewing process. BSG is regularly available in large amounts at a low market price. In 2019, 1.91 billion hectoliters of beer were produced worldwide [1] generating 20 kg of BSG for each hectoliter [2].

BSG consists of the husks that covered the malted barley grains (*Hordeum vulgare*), in mixture with part of the pericarp and seed coat layers that are separated as residual solid material from the liquid wort after the mashing phase of beer production. As a lignocellulosic material, its main constituents are fibers (70% on dry basis) and crude protein (20%) with the remaining portion mainly represented by lipids [3]. According to the type of beer produced, other cereals such as maize, rice, wheat, oats, rye or sorghum can be used in mixture with the barley malt for the wort preparation, thus corresponding to some compositional differences in BSG [4]. 

Several factors limit BSG reuse in the food industry, such as the microbiological instability due to the high moisture (70–80%), the presence of fermentable sugars and the poor technological and sensory characteristics [5,6]. For these reasons, BSG is overall intended as feed and only in small part used to produce biogas [6]. Nevertheless, BSG production often exceeds the demand for local feed, which results in disposal issues in terms of the sustainability and environmental impact of breweries [4]. 

BSG composition can vary, due to the barley cultivar used and harvest time, the presence of other cereal residues (deriving from malted or raw cereals often added to barley malt), and the malting and mashing conditions. Nonetheless, BSG always contains high levels of dietary fiber, proteins, and essential amino acids, as well as appreciable levels of minerals, polyphenols, vitamins, and lipids [7]. Its valuable composition, together with the low cost, make BSG an interesting material that has the potential to be used for the production of goods with high added value. 

Innovative uses of BSG in food production are increasingly being sought by the scientific and food industry communities. BSG-based formulations of cookies, bread, pasta, and snacks with different fortification level have been proposed [8,9,10,11,12], but only few commercial applications are currently available on the global market. Furthermore, antioxidant, anti-atherogenic, anti-inflammatory, and anti-carcinogenic activities of the native chemical compounds of BSG have been shown [7,13,14,15]. Overall, microbial biotechnologies have been reported as essential in BSG utilization in food, feed, pharmaceutical and cosmetic industries [6].

In a previous study [15], it was demonstrated that bioprocessing based on pre-treatment with xylanase and fermentation with selected lactic acid bacteria (LAB) allowed the release of phenolic compounds otherwise bound to the BSG matrix and unavailable for physiological functions. The study highlighted the fundamental role of fermentation in releasing active compounds, maximizing BSG antioxidant potential.

The aim of this study was to evaluate the properties of bioprocessed BSG as ingredient for pasta formulation and to verify its antioxidant properties. A broad-spectrum characterization of the pasta containing BSG including nutritional, technological, structural, and sensory features was performed. The bioaccessibility of the antioxidant compounds, assessed as antioxidant activity towards Caco-2 cells, was investigated on pasta subjected to mimicked gastric digestion.

## 2. Materials and Methods

### 2.1. Raw Materials and Microorganism

The brewers’ spent grain, provided by Peroni (Bari, Italy) brewery, was used in this study. Peroni BSG is obtained from the production of a lager beer brewed with barley malt (70%) and maize (*Zea mays*) (30%) and it does not contain spent yeast. Before any experiment, BSG was grinded with a laboratory mill Ika-Werke M20 (GMBH, and Co. KG, Staufen, Germany). The BSG proximal composition (on dry matter) was: protein, 21%; fat, 10.9%; cellulose, 22.5%; hemicellulose, 25%, lignin, 15.3%; ashes, 5.1%. BSG moisture was 80%. Wheat (*Triticum durum*) semolina was purchased from Selezione Casillo Ltd. (Corato, Italy). 

*Lactiplantibacillus plantarum* (previously *Lactobacillus plantarum*) PU1, belonging to the Culture Collection of the Department of Soil, Plant and Food Sciences (University of Bari, Italy) was used in this study. This strain was previously selected as starter for BSG fermentation based on its kinetics of growth and acidification and the ability to increase antioxidant activity of BSG [15].

The strain was routinely propagated on De Man, Rogosa and Sharpe (MRS) broth (Oxoid, Basingstoke, Hampshire, UK) at 30 °C. Before inoculation it was cultivated until the late exponential phase of growth was reached (ca. 10 h), harvested by centrifugation at 10,000× *g* for 10 min at 4 °C, washed twice in 50 mM sterile phosphate buffer (4 °C, pH 7.0), resuspended in sterile distilled water and used to inoculate BSG. 

The commercial hydrolytic enzyme, Depol™ 761P (Biocatalysts, Chicago, IL), a preparation derived from *Bacillus subtilis* having xylanase activity (14670 nkat/g), was used for the BSG treatment before fermentation. 

### 2.2. BSG Bioprocessing

Fermentation of BSG was carried out as described by Verni et al. [15]. Briefly, BSG was homogenized with water at a 60:40 ratio, supplemented with the enzyme preparation Depol™ 761P (100 nkat/g), and incubated at 50 °C for 5 h. After enzymatic treatment, *L. plantarum* PU1, cultivated as above described, was inoculated (initial cell density ca. 7.5 cfu/g) and the mixture incubated at 30 °C for 24 h. Fermentation was monitored by measuring, before and after incubation, pH and enumerating presumptive LAB using MRS agar medium, supplemented with cycloheximide (0.1 g/L). Plates were incubated in anaerobic condition (AnaeroGen and AnaeroJar, Oxoid) at 30 °C for 48 h. pH values were determined by a pH meter (Model 507, Crison, Milan, Italy) with a food penetration probe. The AACC method 02-31.01 [16] was used for the determination of total titratable acidity (TTA) of samples.

### 2.3. Pasta Making

Two types of pasta containing BSG were made: BSG-p and fBSG-p in which 15% of semolina was replaced by native and fermented BSG respectively. In both cases the BSG was dried and milled before use. Freeze-drying was used to avoid alterations of the biochemical characteristics of BSG before pasta making. BSG was grinded with a laboratory mill Ika-Werke M20 (GMBH, and Co. KG, Staufen, Germany). After milling, the BSG particle size distribution was: 250–500 μm (60%), 500–750 μm (30%), 750–1000 μm (10%). Moisture of the freeze dried BSG and fBSG was 5.85 ± 0.02 and 5.89 ± 0.06% (*w/w*) respectively. A semolina pasta (W-p) without BSG addition was made and used as a control. The proximate composition of wheat semolina used for pasta making was as follows: Moisture, 8.0%; protein, 12.9%; fat, 2.0%.; available carbohydrates, 73.4%, dietary fiber 2.7%.; and ash, 0.9%. Pasta was manufactured using a pilot plant La Parmigiana SG30 (Manufacture, Fidenza, Italy). Table 1 summarizes the ingredients used for pasta making. The doughs had a final dough yield (DY, dough weight × 100/flour weight) of 130, corresponding to 23% (*wt/wt*) water and 77% (*wt/wt*) dry matter (semolina, fBSG or BSG). Ingredients were mixed in three steps (1 min mixing and 6 min hydration). Then, the final dough was mixed for 30 s and extruded at 45–50 °C, through a no. 76 bronze die (die diameter 110mm, holes diameter 7 mm). The extruded material was cut with a rotating knife for short pasta shapes to obtain grooved “macaroni”. For drying, pasta was arranged on frames (1.5 kg per frame) and treated according to the cycle described in Table 1S, at low temperature (55 °C). 

### 2.4. Biochemical and Nutritional Characterization

Chemical and nutritional characteristics were determined on uncooked pasta samples. pH values of BSG-p, fBSG-p and W-p were determined online by a pH meter (Model507, Crison, Milan, Italy) with a food penetration probe. The AACC method 02-31.01 was used for the determination of total titratable acidity (TTA) of experimental pasta. The pH and TTA were determined as described before, after homogenization of the pasta in distilled water. The moisture, protein, lipids, total dietary fiber, and ash of raw materials were determined according to Approved Methods 44-15A, 46-11A, 30-10.01, 32-05.01, and 08-01.01 of the American Association of Cereal Chemists [16].

### 2.5. Technological Characterization 

#### 2.5.1. Optimal Cooking Time

The AAAC approved method 66–50 [16] was used to determine the cooking time. Twenty-five grams of pasta were put into a beaker containing 300 mL of boiling water (without salt addition). Every 30 s, some macaroni pieces were taken out and pressed between two glass plates. The optimal cooking time (OCT) corresponded to the disappearance of the white core [17].

#### 2.5.2. Hydration Test

Five grams of pasta sample were placed in a beaker containing 100 mL of tap water (ratio pasta; water of 1:20), which was placed in a thermostatic bath at 25 °C. After 5, 10, 15, 30, 60, 90 and 180 min of incubation, samples were removed from water, drained for 1 min, carefully blotted with tissue paper to remove superficial water, and weighed. The results were expressed as ((W1 − W0)/W0) × 100, where W1 is the weight of the hydrated sample and W0 is the weight of the dry sample [18].

#### 2.5.3. Cooking Loss and Water Absorption

Cooking loss was evaluated by determining the amount of solid losses into the cooking water [19]. Portions of 30 g of pasta were cooked in 300 mL of boiling tap water (ratio pasta:water of 1:10) without salt addition. Pasta samples were cooked for the OCT. After cooking, the volume of water was brought to the initial volume. Dry matter was determined on 25 mL of freeze-dried cooking water. The residue was weighed, reported as percentage of the dry material, and expressed as grams of matter loss/100 g of pasta. 

The water absorption during cooking was evaluated by weighing pasta before and after cooking at OCT. It was calculated as ((W1 – W0)/W0) × 100, where W1 is the weight of cooked pasta and W0 is the weight of the uncooked samples.

#### 2.5.4. Texture Analysis

Mechanical properties of pasta were analyzed using a texture analyzer, Texture analyzer FRTS-100N, (Imada, Toyohashi, Japan) equipped a 10 kg load cell and a cylinder probe FR-HA-50J. For the analysis, pasta samples were cooked until the OCT, rinsed with water at 25 °C and placed into a beaker (about 30 g), filled to about half volume. The following settings were used: test speed 1 mm/s, 30% deformation of the sample, and two compression cycles. Each measurement was performed in 3 replicates obtained in different batches and the parameters evaluated were hardness (expressed as the maximum force at first compression), cohesiveness (ratio of the areas of the second and the first compression peak), springiness (height of the product on the second compression divided by the height of the first peak), and chewiness (hardness × cohesiveness × springiness).

#### 2.5.5. Color Analysis

The CIELab color space coordinates L (lightness), a* (red-green chromaticity) and b* (yellow-blue chromaticity) of the samples were obtained by a Minolta CR-10 camera and a D65 illuminant. Color difference, ∆E*ab, was calculated as follows:

(1) $$\Delta Eab=\sqrt{\Delta a^{2}+\Delta b^{2}+\Delta L^{2}}$$

where ∆a, ∆b and ∆L are the differences for L, a, and b values between sample and reference (a white ceramic plate having L = 93.4, a = −1.8, and b = 4.4).

### 2.6. Analysis of the Pasta Microstructure 

Pasta samples were cooked in boiling water according to OCT, then transferred to cold water for one minute and drained on a tissue. Samples were cut approximately 10 mm from the edge of the pasta and embedded in PELCO Cryo-Embedding Compound (Ted Pella Inc., Redding, CA, USA) and frozen in liquid nitrogen. Thin cross sections (7 µm) were cut using a Leica CM 1900 cryostat (Leica Microsystem, Wetzlar, Germany) at −20 °C and put on polysine coated microscopy slides.

#### 2.6.1. Brightfield Microscopy

For brightfield microscopy sections were stained with light green and Lugol’s solution respectively as well as a combination of the two. Light green stains proteins green. Lugol’s solution stains starch granules blue/violet, amylose in blue and amylopectin brown/pink. The stained sections were examined using an Olympus BX53 microscope (Olympus Life Science, Tokyo, Japan) and micrographs were captured with a CMos SC50 camera (Olympus Life Science) and processed with the Olympus software cellSense Entry. Pasta samples subjected to the in vitro digestion (see paragraph 2.8.1) were also subjected to the analysis. 

#### 2.6.2. Confocal Laser Scanning Microscopy

For confocal laser scanning microscopy (CLSM) sections were fixed 30 min 4% paraformaldehyde in Phosphate Buffered Saline (PBS) buffer (pH 7.4) and then rinsed with PBS. The sections were preincubated 40 min in PBS Buffer with 2.5% BSA (Bovine Serum Albumin) before applying the primary antibody, LM11 (Plant Probes, Leeds, UK) diluted 1:50 in PBS containing 0.5% BSA, for 2 h. Negative controls were made by replacing the Primary antibody solution with PBS containing 0.5% BSA. After incubation, the sections were rinsed thoroughly with PBS and then incubated for 2 h in the dark with fluorescently labelled secondary antibody Alexa Fluor® 647 (Invitrogen, Carlsbad, CA, USA). All incubations were performed in moisturized chambers at room temperature. Sections were then rinsed with PBS and water and mounted with ProLong®Diamond (Invitrogen, Carlsbad, CA, USA) anti-fading reagent. Micrographs were acquired using a CLSM (Leica TCS SP5, Heidelberg, Germany) a 488 nm argon laser and a 633 nm HeNe laser with a HCX PL APO lambda blue 20.0 × 0.70 IMM UV objective, zoom 1× and 3×. Emissions were collected at 500–550 and 650–700 nm. Images were acquired in the format 1024 × 1024 pixels, 8 lines average.

### 2.7. In Vitro Protein and Starch Digestibility and Nutritional Indexes 

The in vitro protein digestibility (IVPD) of pasta samples, cooked until the OCT, was determined by the method of Akeson and Stahmann [20] modified by Rizzello et al. [21]. The IVPD was expressed as the percentage of the total protein, which was solubilized after enzyme hydrolysis. The modified method of AOAC [22] was used to determine the total amino acid profile of the digested protein fraction [23]. Amino acids were analyzed by a Biochrom 30^+^ series Amino Acid Analyzer (Biochrom Ltd., Cambridge Science Park, United Kingdom), equipped with a Li-cation-exchange column (4.6 × 200 mm internal diameter), as described by De Pasquale et al. [24]. Since the above procedure of hydrolysis does not allow the determination of tryptophan, it was estimated by the method of Pintér-Szakács and Molnán-Perl [25]. Chemical score (CS) estimates the amount of protein required to provide the minimal essential amino acids (EAA) pattern for adults, which was recently re-defined by FAO in 2007 [26]. It was calculated using the equation of Block and Mitchel [27]. The sequence of limiting essential amino acids corresponds to the list of EAA, having the lowest chemical score [27]. The protein score indicates the chemical score of the most limiting EAA present in the test protein [27]. Essential amino acid index (EAAI) estimates the quality of the test protein, using its EAA content as the criterion [28]. EAAI was calculated according to equation:

(2) $$EAAI=\frac{\sqrt{\left(EAA_{1}\times 100\right)\left(EAA_{2}\times 100\right)\left(\ldots \right)\left(EAA_{n}\times 100\right)\left[sample\right]}}{\sqrt{\left(EAA_{1}\times 100\right)\left(EAA_{2}\times 100\right)\left(\ldots \right)\left(EAA_{n}\times 100\right)\left[reference\right]}}$$

The Biological value (BV) indicates the utilizable fraction of the test protein [28]. BV was calculated using equation:

BV = ([1.09 × EAAI] − 11.70)(3)

The protein efficiency ratio (PER) estimates the protein nutritional quality based on the amino acid profile after hydrolysis. PER was determined using equation, developed by Ihekoronye [29]:

PER = − 0.468 + (0.454 × [Leucine]) − (0.105 × [Tyrosine])(4)

The nutritional index (NI) normalizes the qualitative and quantitative variations of the test protein compared to its nutritional status. NI was calculated using equation of Crisan and Sands [30], which considers all the factors with an equal importance:

NI = (EAA × Protein(g/100g) × 100)(5)

The evaluation of the starch hydrolysis rate was performed using a procedure mimicking the in vivo digestion of starch [31]. Wheat flour bread (WB) was used as the control to estimate the hydrolysis index (HI = 100). The predicted GI of all pasta samples was calculated using the following equation [32]:

pGI = 0.549 × HI + 39.71 (6)

### 2.8. Antioxidant Activity and Phenolic Profile

#### 2.8.1. In Vitro Digestion 

Samples of pasta were in vitro digested until the gastric phase according to the INFOGEST method [33]. Briefly, cooked pasta mixed with Simulated Salivary electrolyte Fluid (SSF), at 1.25 × electrolyte solution in a ratio of 1:1 (*w/w*). The resulting mixture was minced to simulate the mastication and then CaCl_2_H_2_O_2_ was added to achieve a concentration of 1.5 mM. The concentration of SSF was adjusted to 1× adding distilled water. The oral bolus was incubated for 2 min at 37 °C under stirring conditions (200 rpm). Gastric electrolyte solution 1.25 × (SGF) was added to the oral bolus in a ratio of 1:1 (vol/vol) and the pH was adjusted to 3 with HCl 5M. Porcine pepsin (Sigma Aldrich, Saint Louis, MO, USA) solution was prepared in water and added to achieve an activity of 2000 U/mL in the final digestion mixture. The concentration of SGF was adjusted to 1× by adding the remaining water. Samples were incubated for 2 h at 37 °C under stirring (200 rpm). Gastric bolus was freeze dried to remove the water for subsequent analysis. 

#### 2.8.2. Extraction of Phenolic Compounds

Cooked pasta was ground and freeze dried to remove the absorbed water. The extraction of the free fraction of phenolic compounds from raw, cooked, and pre-digested pastas was performed with a solvent mixture of acetone/water (4:1). Briefly, five grams of each pasta sample were suspended in 20 mL of the solvent mixture. After 1 h stirring in the darkness, the supernatant was filtered through a cellulose paper. This procedure was repeated twice. Finally, the supernatants were collected and dried by a rotatory evaporator under reduced pressure at 50 °C and recovered with 5 mL of methanol (HPLC grade) [34]. Methanolic extracts were assayed for the 2,2-diphenyl-1-picrylhydrazyl (DPPH) radical scavenging activity [35] and for the analysis of the phenolic compounds.

#### 2.8.3. Caco-2 Cell Culture 

Human colon carcinoma Caco-2 cells (ATCC, HTB-37 clone) were routinely cultured in 25 cm^2^ culture flasks (BD Biosciences, New Jersey, USA) in Dulbecco’s modified Eagle’s medium (DMEM) at pH 7.4. Supplementation was with 10% (*v/v*) heat-inactivated fetal bovine serum (FBS), 1% (*v/v*) HEPES 1M, 1% (*v/v*) non-essential amino acids (Gibco), 1% L-glutamine 200 mM, penicillin (100 U/mL), and streptomycin (100 mg/mL) (Aurogene, Italy). Cells stayed in an incubator at 37 °C in a 5% CO_2_ and 95% air-humidified atmosphere. Refreshing of the medium was every 2–3 days and cells were split once a week. Cells were observed periodically for changes in growth by using an inverted-phase contrast microscope (Olympus, CK2 model). For cell treatments, freeze-dried digested pasta samples were resuspended in DMEM (10 mg/mL, stock solution) and sterilized through 0.22 mm filter membrane (Millipore Corporation, Bedford, MA, USA). 

#### 2.8.4. Detection of Intracellular Reactive Oxygen Species (ROS)

The assessment of the level of ROS was carried out by measuring the oxidation of the Cell Rox green probe (Thermofisher scientific, USA). Briefly, the confluent Caco-2 cells in the 96-well plates underwent pre-treatment for 18 h with freeze-dried digested pasta samples (100 µg/mL). Menadione (50 µM) was added 2 h before the end of the incubation to induce oxidative stress. Then, cells were stained with 5 µM of Cell Rox green probe and were incubated for 30 min at 37 °C. After washing with PBS, cells were fixed in 3.7% formaldehyde/PBS and permeabilized with 0.5% Triton X-100 for 15 min and analysed by fluorescent microscopy FLoid™ Cell Imaging Station. The fluorescence intensity (relative fluorescence units) was measured (Spectrofluorimeter Victor3, Perkin-Elmer, Waltham, MA, USA) at an excitation and emission wavelength of 485 nm and 520 nm, respectively. 

#### 2.8.5. Catalase Activity

Seeding of Caco-2 cells was on 6-well plates. After differentiation (14–15 days), cells underwent treatment for 3 h with freeze-dried digested pasta samples and subsequent stimulation for 30 min with Menadione 50 µM (positive control). Catalase activity was determined according to the method described by Li and Schellhorn [36], based on the determination of the hydrogen peroxide decomposition rate. After treatment, the cells were collected and incubated for 30 min in iced lysis buffer [1 mM EDTA, 1 mM phenylmethanesulfonyl fluoride (PMSF, Sigma), 0.1% *v*/*v* Triton X-100 and 1 mM dithiothreitol (DTT, Sigma)]. After centrifugation (11,000× *g* for 4 min), the supernatant was separated and catalase activity was determined and normalized per mg of protein. Catalase assay was performed with 230 µL of phosphate buffer (NaH2PO4, 0.05 M, pH 7.0), 10 µL of cell lysate and 10 µL of Menadione at the final concentration of 20 mM. Spectrophotometric reading was immediate at 240 nm every 10 s for 3 min. 

#### 2.8.6. Assessment of Intracellular Reduced (GSH) and Oxidized (GSSG) Glutathione

Confluent Caco-2 cells in the 6-well plates underwent pretreatment for 6 h with freeze-dried digested pasta samples (100 mg/mL), were stimulated with a mixture of INF- γ (8000 U/mL) and PMA (0.1 mg/mL) for 18 h. At the end of the treatment, cells were washed three times with PBS, and homogenized with 200 μL of buffer (50 mM phosphate buffer, pH 7, containing 1 mM EDTA and 0.5% NP40) added to each well, and then cells were collected using a rubber spatula. Cells were freeze−thawed three times between −80 °C and room temperature (RT) and deproteinized with an equal volume of metaphosphoric acid (10%, *w/v*). Thereafter, cell lysates were incubated at RT for 5 min and separated by centrifugation (10,000× *g*, for 10 min at 4 °C). The supernatant was decanted for GSH and total GSH−GSSG quantification using the GSH/GSSG Ratio Detection Assay Kit (Abcam, Cambridge, U.K.) according to the manufacturer’s instructions. Finally, the fluorescence was measured (λex/em = 490/520 nm) using a Spectramax fluorescent plate reader. GSH and GSSG concentrations were calculated using a calibration curve obtained with dilutions of pure GSH and GSSG (Merck, Darmstadt, Germany). The GSH/GSSG ratio was calculated by dividing the difference between the GSH and GSSG concentrations by the concentration of GSSG.

### 2.9. Sensory Analysis

Sensory evaluation of cooked pasta was carried out at the sensory laboratory of University of Helsinki, which fulfils the requirements of the ISO standards [37,38]. A descriptive sensory analysis method was used to define the main sensory attributes and their intensities in the samples. The study was carried out by 10 trained panelists (4 male and 6 female, mean age: 30 years, range: 20–45 years). Sensory attributes and related reference standards, discussed and agreed with the assessors during the three sensory training sessions, are listed in Table S2. A line scale from 0 to10, with 10 indicating very high intensity of a sensory attribute, was used to rate the attributes. The evaluations were performed at room temperature at individual speeds in separate booths using the FIZZ sensory analysis software (version 2.47, Biosystèmes, Couternon, France). The samples were cooked (according to their OCT, Table 3) and presented randomized (Latin square) in triplicate. The mouth was rinsed with tap water between the samples. A commercial pasta at high fiber content, made with whole wheat (Pasta di Stigliano, Italy), was included in the study. The study protocol followed the ethical guidelines of the sensory laboratory of Department of Food and Nutrition, University of Helsinki, that has been accepted by The University of Helsinki Ethical Review Board in the Humanities and Social and Behavioral Sciences (Statement 46/2016). A written informed consent was obtained from each participant.

### 2.10. Statistical Analysis

All the analysis were carried out in triplicate. The one-way ANOVA, using Tukey’s procedure at *p* < 0.05, was performed for the data elaboration (Statistica 12.5, StatSoft Inc., Tulsa, OK, USA).

## 3. Results

### 3.1. Native and Bioprocessed BSG 

During BSG fermentation, the value of pH decreased from 4.98±0.15 (T0) to 3.75 ± 0.11 (T24), with TTA values respectively of 3.59 ± 0.21 and 12.72 ± 0.62 mL NaOH 0.1 M after 24 h. LAB cell density of the bioprocessed BSG was 8.62 ± 0.34.

### 3.2. Biochemical and Nutritional Characteristics of the Pasta Samples

Compared to the semolina pasta W-p, pasta containing BSG had a significantly lower pH (Table 2). BSG is indeed characterized by a lower pH compared to semolina dough (4.98 ± 0.15), that further deceased during fermentation to 3.75 ± 0.11 in fBSG, as the consequence of the organic acids synthesis by lactic acid bacteria. Indeed, the lowest value was found for fBSG-p, also having a TTA value more than 4- and 3-times higher than W-p and BSG-p, respectively. 

No significant (*p* < 0.05) differences were found in the proximal composition of BSG-p and fBSG-p. Nevertheless, the high content of proteins and fibers of BSG reflected in both the fortified pasta samples compared to W-p, independently of the bioprocessing (Table 2). In details, the BSG fortification led to increase in proteins up to 7%, and a fibers concentration ca. 4-times higher than W-p (Table 2). The fat content was significantly higher in pasta samples containing BSG. On the contrary, available carbohydrates were the highest in W-p (Table 2).

### 3.3. Technological Characterization of the Pasta Samples

**Figure 1:**
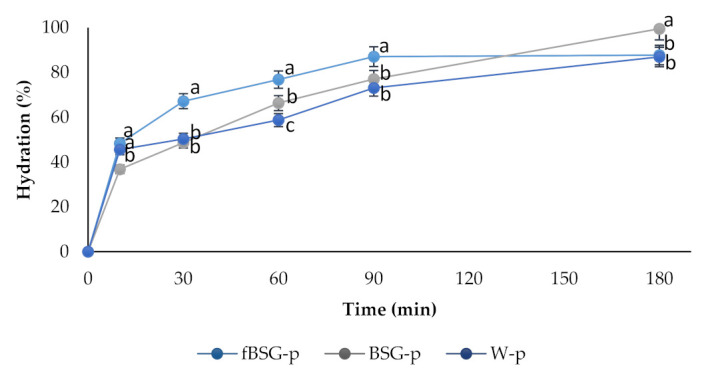
Kinetics of water absorption of pasta at 25 °C. BSG-p, pasta containing native brewers’ spent grains (15% *wt/wt* in replacement of semolina); fBSG-p, pasta containing fermented brewers’ spent grains (15% *wt/wt* in replacement of semolina); W-p, pasta made with durum wheat semolina. Data are the means of three independent analyses. ^a–c^ Values with different superscript letters within the same time, differ significantly (*p* < 0.05). Bars of standard deviations are also represented.

The kinetics of water uptake at 25 °C are shown in Figure 1. The sample of pasta containing fermented BSG (fBSG-p) was characterized by a faster water uptake as compared to BSG-p and W-p.

The experimental OCT of W-p was 9 min. A 3 min decrease in OCT for pasta containing native or bioprocessed BSG was found (Table 3). Water absorption (retained during cooking) decreased as the consequence of BSG addition, especially in fBSG-p (−22% compared to W-p). Cooking loss was significantly (*p* < 0.05) higher in fBSG and BSG-p compared to the semolina control and increased by 77 % when fermented BSG was used as ingredient. The instrumental textural properties of the pasta samples were investigated on cooked samples until OCT. W-p had the lowest value of hardness. The presence of BSG increased the hardness of ca 49% in BSG-p and 11% in fBSG-p (Table 3). Compared to W-p, chewiness decreased with the fortification, with the lowest value found for fBSG, while springiness and cohesiveness were not affected (Table 3). As a result of fortification with BSG, pasta color showed a different profile (Table 3), characterized by lower lightness and higher red index (a). Significant differences in lightness were moreover observed between BSG-p and fBSG-p, this latter characterized by a significantly (*p* < 0.05) higher value (ca. 12%). 

### 3.4. Microstucture Characterization

**Figure 2:**
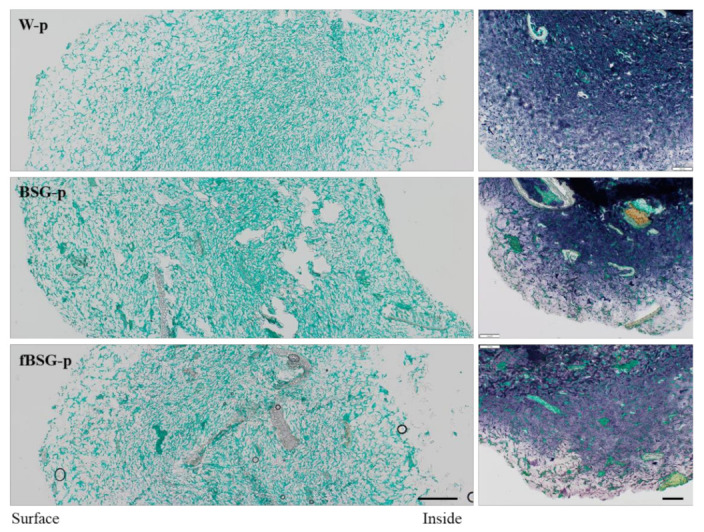
Brightfield microscopy analysis of pasta containing fermented and native brewers’ spent grain: BSG-p, pasta containing native brewers’ spent grain (15% *wt/wt* in replacement of semolina); fBSG-p, pasta containing fermented brewers’ spent grain (15% *wt/wt* in replacement of semolina); W-p, pasta made with durum wheat semolina. Proteins are shown in green and starch is shown in blue. Fibers are unstained. Scale bar left panel 200 µm and right panel 100 µm.

All samples showed gradual microstructural changes from the surface to the middle of the pasta wall. As expected, pasta cooking led to the formation of an outer zone of strongly swollen and partly disintegrated starch granules (Figure 2, right panels), and the phenomenon is more evident in both BSG containing pasta. The degree of starch swelling decreased towards the core. The leaching of amylose (dark blue) was observed in all the samples. Microscopy analysis of the pasta samples revealed a continuous protein network (green) in all samples (Figure 2, left panels), although BSG supplementation led to a heterogeneous protein structure. Indeed, protein aggregates of different sizes deriving from BSG were integrated in the wheat gluten network. In BSG-p, part of the BSG proteins remained trapped inside the aleurone cells or connected to the sub-aleurone layer, while a more homogeneous distribution of the BSG proteins throughout the gluten network was observed in fBSG-p. 

**Figure 3:**
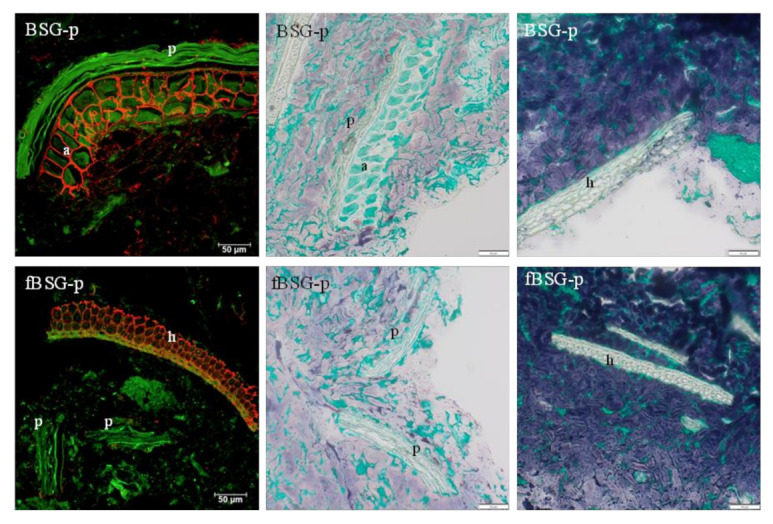
CLSM (left) and brightfield (middle, right) micrographs of pasta containing fermented and native brewers’ spent grain: BSG-p, pasta containing native brewers’ spent grain; fBSG-p, pasta containing fermented brewers’ spent grain. In CLSM micrographs arabinoxylan is shown in red and autofluorescence (cell walls, protein) is shown in green. Bright field micrographs show starch in blue and protein in green. h= hull (palea and lemma), a = aleurone, p = pericarp. Scale bars 50 µm.

**Figure 4:**
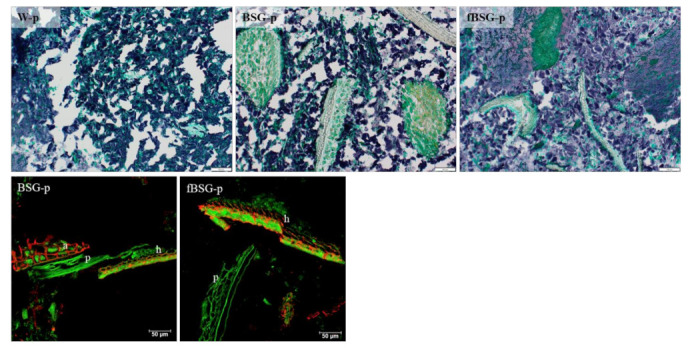
Brightfield and CLSM microscopy analysis performed on the in vitro-digested pasta containing fermented and native brewers’ spent grain: BSG-p, pasta containing native brewers’ spent grain; fBSG-p, pasta containing fermented brewers’ spent grain. W-p, pasta made with durum wheat semolina. Upper row: Bright field micrographs. Proteins are stained green and starch is stained blue. Scale bar 100 µm. Lower row: CLSM micrographs. Arabinoxylan is shown in red and autofluorescence (cell walls, protein) in green. h = hull (palea and lemma), a = aleurone, p = pericarp. Scale bars 50 µm.

CLSM micrographs (Figure 3) confirmed that the BSG-derived aleurone cells in BSG-p were characterized by an intact and tight arabinoxylan structure. In the hull (palea and lemma) the cell walls also stained positive for arabinoxylan, both in BSG-p and fBSG-p. In fBSG-p, only few intact aleurone cells were observed, although remains of the pericarp and parts of the hull poorly affected by the treatment were still detectable, shown in both CLSM and brightfield micrographs. The brightfield micrographs details reported in Figure 3 moreover showed that BSG-derived fibers were neatly embedded in the starch-protein matrix. Brightfield microscopy analysis performed on the in vitro-digested pasta (Figure 4) showed that the in vitro digestion led to an extensive breakdown of the protein network. Large protein structures originated from BSG characterized the fortified pasta. The in vitro digestion did not affect the structure of the aleurone cells, that were in large part intact, with all the proteins inside, nor the palea and lemma. 

### 3.5. Protein and Starch Digestibility and Nutritional Indexes

Similar protein digestibility characterized W-p and BSG-p, while fBSG-p had a significantly (*p* < 0.05) higher IVPD value (+16%) (Table 4). Based on CS, the sequence of limiting amino acids for all the pasta samples was found to be Lys, Thr, and His; nevertheless, the CS value for Lys and His were significantly higher in fBSG-p compared to the other pasta samples. Compared to W-p, the CS for His, Ile and Leu of fBSG-p were respectively 10, 32 and 26% higher. EAAI and BV were significantly (*p* < 0.05) higher for fBSG-p compared to W-p and BSG-p, which showed instead similar values for both the indexes (Table 4). The same was observed for NI, that resulted as almost 2-times higher in fBSG-p than BSG-p and W-p (Table 4). The starch hydrolysis of the pasta samples was markedly affected by BSG addition. Compared to white bread, used as the analytical control and corresponding to HI = 100, the HI value of W-p was 68.65% and significantly (*p* < 0.05) lower values were found for BSG-p (63.46%) and fBSG-p (58.86%). As a result, the predicted GI value of fBSG-p was the lowest (Table 4).

### 3.6. Total Phenols and Antioxidant Activity

#### 3.6.1. Total Phenols and Radical Scavenging Activity

The analysis of the total phenols was carried out on raw, cooked (at OCT) and in vitro-digested pasta. Raw or cooked BSG-p and fBSG-p showed a significantly (*p* < 0.05) higher total concentration of free phenolic compounds than W-p (Table 5). However, fBSG-p did not show a significant (*p* > 0.05) difference compared to BSG-p. Cooking did not significantly affect total phenolic content in any case (Table 5). However, gastric digestion significantly (*p* < 0.05) released phenolic compounds in all pasta samples, which were more abundant in BSG-p and fBSG. In addition, the supplementation of pasta with fermented BSG (fBSG-p) significantly enhanced the release of phenolic compounds during the gastric digestion compared to BSG-p (Table 5). 

Almost the same trend for the antioxidant activity, assayed as radical scavenging activity towards DPPH radical, was found. BSG-p and fBSG-p, raw or cooked, showed a higher radical scavenging than semolina control. Nevertheless, fBSG-p had a significantly (*p* < 0.05) higher activity than BSG-p both cooked and pre-digested (Table 5). Overall, cooking caused a significant decrease of the antioxidant activity in all the samples while mimicked digestion increased radical scavenging in all pasta samples, despite significant differences among samples. In particular, pre-digested fBSG-p showed antioxidant activity 15 and 23% higher than BSG-p and W-p, respectively.

#### 3.6.2. Antioxidant Features of fBSG-p in Caco-2 Cell Culture 

**Figure 5:**
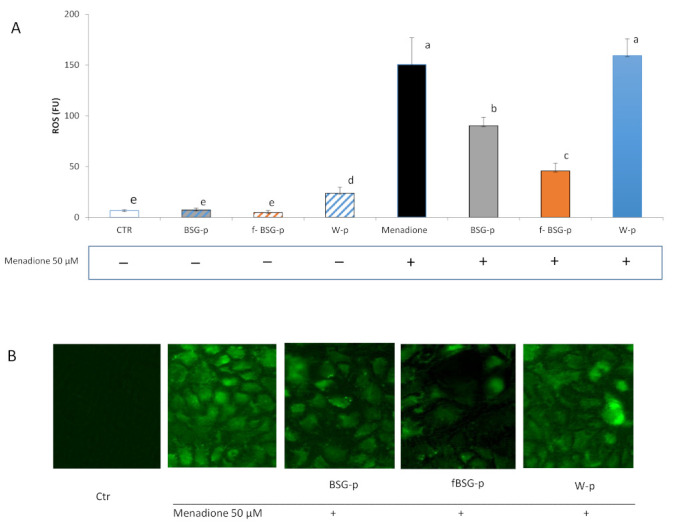
Intracellular reactive oxygen species (ROS) (fluorescence intensity units, FU) (**A**) and representative fluorescent microscopic images (**B**). Green fluorescent signal denotes ROS detection by the CellROX® Green reagent. ROS induction was detected utilizing CellROX® Green reagent coupled with the SpectraMax® MiniMax™ Imaging cytometer as described in the Materials and Methods. Caco-2 cells were treated with wheat pasta (W-p), pasta containing native (BSG-p), and fermented (fBSG) brewers’ spent grain (BSG) or not treated (Ctr). To induce oxidative stress, Caco-2 cells were treated with 50 µM Menadione. Data are the means (± SD) of three independent experiments analyzed in triplicate. Data were subjected to one-way ANOVA followed by Tukey’s procedure at *p* < 0.05. Bars with different superscript letters differ significantly (*p* < 0.05).

The potential protective activity of the pasta samples towards oxidative stress was investigated on human colon carcinoma cells (Caco-2). Caco-2 cells were treated with freeze-dried and digested W-p, BSG-p, and fBSG-p for 18 h, and 2 h before the end of the incubation an oxidative stress was induced by menadione addition. ROS were detected utilizing a fluorescent reagent (Figure 5A,B). The lowest (*p* < 0.05) level of intracellular ROS was detected when cells were incubated in presence of the digested fBSG-p. In particular, ROS were ca. 70% lower than those found in Caco-2 cells treated with W-p. Additionally, the treatment with BSG-p led to a significant decrease (ca. 35%) of the ROS generation, compared to W-p. No significant (*p* > 0.05) differences were observed for the W-p treatment compared to the control, in which cells were subjected to the oxidative stress by menadione in medium alone (Figure 5A). The fluorescent microscopic observation confirmed the decrease of ROS level occurring after the BSG-p and fBSG-p treatments (Figure 5B). 

**Figure 6:**
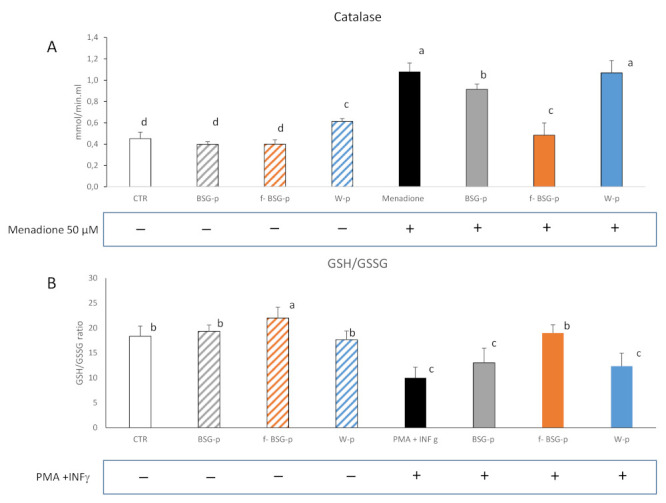
Catalase activity (**A**) and ratio of reduced and oxidized glutathione (GSH/GSSG) (**B**). Caco-2 cells were treated with wheat pasta (W-p), pasta containing native (BSG-p), and fermented (fBSG) brewers spent grain (BSG). To induce oxidative stress, Caco-2 cells were treated with a mixture of PMA INF- γ (8000 U/mL) and PMA (0.1 mg/mL). Data are the means (± SD) of three independent experiments analyzed in triplicate. Data were subjected to one-way ANOVA followed by Tukey’s procedure at *p* < 0.05. Bars with different superscript letters differ significantly (*p* < 0.05).

As expected, the menadione addition markedly increased (*p* < 0.05) the catalase activity of the Caco-2 cells compared to the untreated Ctr (Figure 6A), and the treatment with W-p did not significantly affect the result. Conversely, the presence of digested fortified pasta significantly (*p* < 0.05) counteracted the increase, especially when fBSG-p was used. In particular, the decrease of the catalase activity resulted of 20 and 57% respectively for treatments with BSG-p and fBSGp compared to that with W-p. The scavenging capacity induced by the fBSG-p treatment was also confirmed by the increased ratio of reduced to oxidized glutathione (GSH/GSSG) (Figure 6B), that resulted as 36% and 48% higher than that observed for the BSG-p and W-p treatments. 

### 3.7. Sensory Analysis

**Figure 7:**
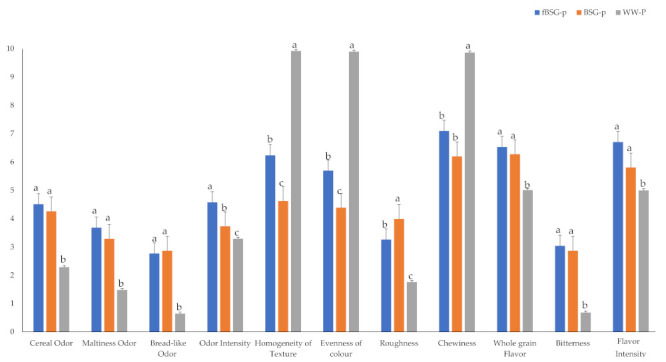
Sensory analysis of pasta containing fermented and native brewers’ spent grain: BSG-p, pasta containing native brewers’ spent grain; fBSG-p, pasta containing fermented brewers’ spent grain; WW-p, commercial pasta made with whole wheat (Pasta di Stigliano, Italy). The 11 sensory attributes were evaluated (on a scale 0–10, Y axis) by a sensory panel of 10 trained assessors in triplicate. Data were subjected to one-way ANOVA followed by Tukey’s procedure at *p* < 0.05. Bars with different letters differ significantly (*p* < 0.05).

Experimental pasta was subjected to descriptive sensory analysis and the results are showed in Figure 7. Descriptive analyses are generally used when detailed specification of the sensory attributes of products and a comparison of the sensory differences among them are desired [39]. The eleven sensory attributes selected during training sessions are listed in Table S2. In this regard, the presence of BSG led panelists to select cereal odor, malt odor, sourdough bread-like odor and bitter taste among the main attributes. As previously reported [40], the acidity of the pasta including fermented ingredients was markedly attenuated by cooking, due to the dilution of the organic acids produced during fermentation, thus making unperceivable the acidic taste.

Commercial pasta made with whole wheat (WW-p) was included as reference in this analysis, since its fiber content is similar to BSG-containing pasta.

Compared to WW-p, the attributes that mainly affected BSG pasta sensory quality were related to aesthetical aspect, such as homogeneity of texture, evenness of color, roughness and chewiness as technological parameter. In particular, WW-p was characterized for the highest texture homogeneity, evenness of color and perceived chewiness (Figure 7). Bitterness perceived in cooked pasta containing BSG, although higher than WW-p, resulted very low (scores of 3 and 1, respectively). 

Compared to BSG-p, the fermentation of BSG in fBSG-p increased the perception of some of the sensory attributes judged with relatively low scores in pasta with unprocessed BSG. In particular, the overall odor intensity, homogeneity of texture and evenness of color were significantly (*p* < 0.05) higher in fBSG-p compared to BSG-p. 

## 4. Discussion

The reorientation of production processes by pursuing solutions towards “zero waste” operations, with subsequent closure of cycles, can strongly contribute to a reduction in agro-industrial waste, maximizing the conversion of feedstock and its by-products, side streams and surplus into higher added-value products [41]. The implementation of environmentally friendly and sustainable technologies, such as fermentation with lactic acid bacteria, is now considered a promising pathway to valorize agri-food side-streams [42]. The scientific community has emphasized the enormous potential in nutritional and functional terms of brewers’ spent grain [4,6,43,44]. In our previous study bioprocessing including xylanase treatment followed by fermentation with selected lactic acid bacteria increased the antioxidant properties of BSG thanks to the release of phenolic compounds otherwise bound to the BSG matrix and unavailable for physiological functions, and of antioxidant peptides [6,15]. In this study, the role of bioprocessed BSG as functional food ingredient has been investigated.

Pasta is traditionally manufactured only using durum wheat semolina; nevertheless, the use of non-wheat flour to increase its nutritional value has been studied [45]. Nowadays, pasta manufactured with different ingredients is available on the market.

To understand the effect of bioprocessing on BSG and consequently on the final food product, pasta containing native or bioprocessed BSG were compared. BSG was added at 15% *wt/wt* in replacement of semolina, based on the pasta technological and sensory acceptability determined in preliminary tests and our previous study on the use of bioprocessed cereal by-products [40]. Moreover, it was recently reported that the addition of native BSG at percentages lower than 20% minimally affected sensory properties of cooked pasta, identifying the semolina replacement of 10% as the best compromise in terms of technological, nutritional, and sensory pasta features [10,44]. The high content of proteins and fibers of BSG reflected in both the fortified pasta samples compared to the conventional semolina control, independently of the bioprocessing. According to EC Regulation [46] on nutrition and health claims on food products, our experimental fortified pasta can be labelled as a “high fiber” and a “source of protein”. The properties of the alternative ingredients used in replacement (albeit partial) of semolina have an enormous impact on the final pasta quality. Fibers interfered with the gluten network development leading to a weaker protein network, with negative consequences on the BSG-containing pasta, such as shorter OCT, lower water absorption during cooking, and higher cooking loss compared to the conventional semolina pasta [10,44,47,48]. The weakening of the gluten network, as well as the processing conditions affected the micro and macro structure of the pasta (porosity) leading to a faster water diffusion and hence an earlier gelatinization during cooking which explains the shorter OCT [47,48]. The decrease of the OCT and the lower water absorption were already reported for pasta supplemented with high amount of BSG [44]. Moreover, cooking loss was higher in pasta made with fBSG, as the result of the partial hydrolysis of proteins and carbohydrates during fermentation that led to the release of soluble components. The textural features of cooked pasta were investigated with an instrumental analyzer. Texture attributes were affected by the inclusion of non-wheat ingredients, resulting in higher hardness and lower chewiness values [40]. Nevertheless, fermentation of the BSG mitigated the hardness increase. This might be due to a more homogeneous distribution of BSG proteins inside the gluten network. 

Indeed, the microscopic analysis showed the BSG aleurone structure almost intact and rich in arabinoxylan in BSG-p and extensively degraded in fBSG-p, thanks to the xylanase activity, as previously reported [15], but the arabinoxylans in the hull had withstand the degradation. The lack of aleurone degradation, whose cells were still enclosing proteins inside their compartments, affected the protein distribution inside the gluten network and thereby the accessibility. In fact, in fBSG-p more BSG-proteins were released and homogeneously distributed inside the protein network. Although well integrated in the surrounding matrix, the high fiber content and the non-gluten proteins weakened the pasta protein network in BSG containing samples. As consequence, the starch swelling increased, affecting the OCT and cooking loss. 

According to literature data [44], the color of pasta containing BSG showed a different profile, characterized by lower lightness and higher red index (a). In the case of products containing fiber, the dark coloring is a positive trait, as consumers associate it with high-fiber products [49]. 

Generally, fermentation has an important role in improving sensory characteristics [50]. Descriptive analysis is generally used when detailed specification of sensory attributes in products for their comparison is desired [39]. Cereal/malt odor and the bitterness were chosen as sensory attributes to describe the characteristics of BSG. Being the fibers largely responsible of the sensory and technological properties of the BSG containing pasta, a commercial wholewheat pasta containing 7% of fibers was included as control. Previous results reported a good acceptability of pasta supplemented with native BSG at percentages lower than 20% [44]. In this work, we demonstrated that fermentation of BSG allowed higher perceived homogeneity, chewiness, and roughness of the pasta, conferring a more intense odor, compared to the use of untreated BSG. 

The protein quality of food depends on several factors such as the essential amino acid ratio, digestibility, and presence of antinutritional factors which further affects the amino acids bioavailability [51]. Proteolysis occurring during LAB fermentation leads to a progressive hydrolysis of native proteins and to the increase of peptides and free amino acids concentration [45]. As consequence, an increase of digestibility of the protein fraction and high nutritional indexes are commonly associated with LAB-fermented foods [52]. This phenomenon reflected in higher IVPD, as well as in higher nutritional indexes of the fermented BSG containing pasta. The NI, combining qualitative and quantitative factors, and considered a global predictor of the protein quality [23,30], resulted almost 2-times higher in fBSG-p than BSG-p and W-p.

The addition of enzymes (i.e., carbohydrases) has been used to increase the amount of soluble fibers in whole grain foods. Fiber solubilization in cereal matrixes, in particular, was as achieved using xylanases [53,54] that, acting on arabinoxylans, allow the solubilization of the fibers insoluble fraction. Our previous results showed that, compared to the untreated, BSG bioprocessed with LAB and xylanase had higher content of soluble fibers [15]. The increase of water-extractable arabinoxylans was correlated with the delay of carbohydrate digestion and absorption rates and with a decrease of the glycemic and insulinemic responses [55,56,57]. Indeed, the HI value of W-p was significantly higher than fBSG-p, and consequently the predicted GI value of fBSG-p was ca. 7% lower than the semolina control.

BSG is recognized as a carrier of valuable phenols whose antioxidant, antiradical, but also anti-carcinogenic and anti-apoptotic properties, have been acknowledged [7]. Most of the phenolic compounds in BSG are hydroxycinnamic acids, of which ferulic acid is the most abundant and strongly correlated with cereal antioxidant capacity. However, since ferulic acid is bound to the ligninocellulosic structure of BSG, it has low bioaccessibility [58]. 

Previously, bioprocessed BSG exhibited enhanced antioxidant activity, characterized by high radical scavenging activity, long-term inhibition of linoleic acid oxidation and protective effect toward oxidative stress on human keratinocytes [15]. The supplementation of fermented BSG to pasta significantly enhanced the phenolic compounds concentration, especially after mimicked gastric digestion. Cooking led to a decrease of the antioxidant activity, as determined by DPPH, while digestion amplified the radical scavenging activity. Whilst some of the peptides and phenolic compounds responsible for the scavenging properties against DPPH might have leached in the cooking water, it is also known that food processing affects its antioxidant properties [59]. Indeed, the presence of oxygen and water triggers the lipoxygenase reaction [60,61], therefore inducing an oxidative degradation of phenolic compounds and the decrease of the antioxidant activity of pasta [59,62]. Cooking methods have an essential role on the stability of phenolic compounds and their subsequent release during digestion. It is worth to note, however, that the fate of phenolic compounds during heat treatments varies greatly. On one hand, it was previously found that some hydroxycinnamic acids, as well as procyanidins and anthocyanins, are highly susceptible to thermal degradation [63,64,65]. On the other hand, heat treatments are of paramount importance for the release of phenolic compounds from food matrices during digestion, consequentially increasing their bioaccessibility [63]. Cooking can promote the release or the hydrolysis of phenolic compounds from the food macromolecules through a matrix softening effect [63,64,65]. In this study, pasta samples cooked and pre-digested up to the gastric phase according to the INFOGEST method [33], were characterized by a higher content of phenolic compounds compared to the undigested pasta. The acidic conditions during the digestion, as well as the presence of enzymes, are most likely responsible for the release of bound phenolic compounds. In fact, most of the phenolic compounds in BSG, and cereals in general, are covalently bounded to cell wall structural components such as cellulose, hemicellulose, lignin, pectin and proteins and alkaline and acidic hydrolyses are the most common means of releasing them [66]. Acid treatment breaks glycosidic bonds leaving ester bonds intact [67], whereas carbohydrate-hydrolyzing enzymes disintegrate the plant cell wall matrix, facilitating polyphenol extraction [66]. Hence, it is likely that the simulated digestion, enhanced by the softening effect of cooking, is responsible for the increase in phenolic compounds in fBSG-p. 

The capability of fBSG-p to protect from oxidative stress was demonstrated on human colon carcinoma cells (Caco-2), widely used as in vitro model of the intestinal mucosa. Intestinal cells are extremely susceptible to oxidative injury when exposed to lipid peroxidation products and luminal oxidants derived by food ingestion [68]. Under the experiment conditions, in which an oxidative stress was artificially induced, the treatment with digested fBSG-p significantly decreased the generation of intracellular ROS, even though BSG-p also conferred a relevant effect in ROS reduction. It can be hypothesized that the enhanced biological availability of phenolic compounds increased the scavenging capacity of the cell culture system and lowered the catalase activity. Indeed, the lowest catalase activity was detected in cells subjected to the treatment with fBSG-p. A further confirmation of the scavenging capacity exerted by fBSG-p on Caco-2 cells was provided by the increased ratio of reduced to oxidized glutathione compared to the other treatments. GSH represents a marker of oxidative stress in inflammatory diseases. It is involved in ROS detoxification and therefore its increase corresponds to an effective limitation of the cellular oxidative damage [69].

## 5. Conclusions

Bioprocessing with xylanase and selected lactic acid bacteria improved the nutritional and functional potential of BSG, through the release of proteins and phenolic antioxidant compounds entrapped into the fiber structure. The detrimental effect of BSG addition to pasta on technological and sensory properties was mitigated by applying bioprocessing as pre-treatment. To contribute to sustainable development, focus on resource management and waste reduction is required that includes valorizing by-products. In this perspective, food material still suitable for food consumption should be kept as close as possible to the food chain [70]. Besides the evident advantages related to BSG bioprocessing, the proposed strategy can be considered an effective and sustainable option for the food sector thanks to: i) the worldwide large availability of BSG, produced both at artisanal and industrial level; ii) the use of commercial food-grade enzymes already on the market at affordable cost; iii) the use of a sourdough-type fermentation, already employed at industrial level and easily applicable in breweries or cereal processing companies, and iv) the economic added value that pasta with the functional status could acquire.

## Figures and Tables

**Table 1:** Formulas for pasta containing fermented and native brewers’ spent grain (BSG): BSG-p, pasta containing native brewers’ spent grain (15% *wt/wt* in replacement of semolina); fBSG-p, pasta containing fermented brewers’ spent grain * (15% *wt/wt* in replacement of semolina); W-p, pasta made with durum wheat semolina.

	BSG-p	fBSG-p	W-p
Semolina (g/100g)	65.4	65.4	77
Water (g/100g)	23	23	23
BSG (g/100g)	11.6	11.6	

* BSG homogenized with water at a 60:40 ratio was added by Depol (100 nkat/g dough) and incubated at 50 °C for 5 h. After enzymatic treatment, *Lactiplantibacillus plantarum* PU1, cultivated as above described, was inoculated (initial cell density ca. 7.5 cfu/g) and incubated at 30 °C for 24 h.

**Table 2:** Biochemical properties of pasta containing fermented and native brewers’ spent grain: BSG-p, pasta containing native brewers’ spent grain (15% *wt/wt* in replacement of semolina); fBSG-p, pasta containing fermented brewers’ spent grain (15% *wt/wt* in replacement of semolina); W-p, pasta made with durum wheat semolina.

	BSG-p	fBSG-p	W-p
**pH**	5.82 ± 0.01 ^b^	5.55 ± 0.04 ^c^	6.93 ± 0.00 ^a^
**TTA**	9.00 ± 0.01 ^b^	12.45 ± 0.05 ^a^	3.00 ± 0.01 ^c^
**Protein (%)**	15.16 ± 0.19 ^a^	14.96 ± 0.25 ^a^	14.11±0.27 ^b^
**Fat (%)**	3.49 ± 0.10 ^a^	3.36 ± 0.08 ^a^	2.18 ± 0.20 ^b^
**Available carbohydrates (%)**	67.47 ± 0.81 ^b^	65.69 ± 0.99 ^b^	79.44 ± 1.01 ^a^
**Total dietary fibers (%)**	11.88 ± 0.09 ^a^	11.96 ± 0.13 ^a^	2.91 ± 0.10 ^b^
**Ash (%)**	1.58 ± 0.07 ^a^	1.62 ± 0.09 ^a^	0.95 ± 0.05 ^b^

Data are expressed % of dry matter. The data are the means of three independent experiments ± standard deviations (*n* = 3). ^a–c^ Values in the same row with different superscript letters differ significantly (*p* < 0.05).

**Table 3:** Technological and structural properties and color indexes of pasta containing fermented and native brewers’ spent grain: BSG-p, pasta containing native brewers’ spent grain (15% *wt/wt* in replacement of semolina); fBSG-p, pasta containing fermented brewers’ spent grain (15% *wt/wt* in replacement of semolina); W-p, pasta made with durum wheat semolina.

	BSG-p	fBSG-p	W-p
*Technological properties*			
OCT (min)	6.2 ± 0.2 ^b^	6.0 ± 0.1 ^b^	9.0 ± 0.1 ^a^
Water Absorption (%)	103 ± 1 ^b^	93 ± 7 ^c^	124 ± 3 ^a^
Cooking loss (% of d.m.)	4.8 ± 0.1 ^b^	6.9 ± 0.3 ^a^	3.9 ± 0.2 ^c^
*Structural properties*			
Hardness (N)	6.33 ± 0.69 ^a^	4.77 ± 0.31 ^b^	4.23 ± 0.21 ^c^
Springiness	0.73 ± 0.12 ^a^	0.71 ± 0.09 ^a^	0.70 ± 0.05 ^a^
Chewiness (N)	2.84 ± 0.12 ^b^	2.41 ± 0.17 ^c^	3.13 ± 0.09 ^a^
Cohesiveness	0.65 ± 0.03 ^a^	0.70 ± 0.02 ^a^	0.68 ± 0.03 ^a^
*Color analysis*			
L	49.53 ± 1.72 ^c^	56.55 ± 5.12 ^b^	69.09 ± 1.71 ^a^
a	3.23 ± 0.58 ^a^	2.72 ± 0.06 ^b^	0.35 ± 0.03 ^c^
b	11.51 ± 0.41 ^c^	12.61 ± 0.18 ^b^	16.70 ± 0.27 ^a^
ΔE	44.74 ± 1.43 ^a^	38.04 ± 4.94 ^b^	27.33 ± 1.63 ^a^

The data are the means of three independent experiments ± standard deviations (*n* = 3). ^a–c^ Values in the same row with different superscript letters differ significantly (*p* < 0.05).

**Table 4:** In vitro protein and starch digestibility and nutritional indexes of pasta containing fermented and native brewers’ spent grain: BSG-p, pasta containing native brewers’ spent grain (15% *wt/wt* in replacement of semolina); fBSG-p, pasta containing fermented brewers’ spent grain (15% *wt/wt* in replacement of semolina); W-p, pasta made with durum wheat semolina.

	BSG-p	fBSG-p	W-p
Nutritional indexes			
In Vitro Protein Digestibility (IVPD) %	78.65 ± 1.03 ^b^	88.52 ± 0.05 ^a^	76.22 ± 1.01 ^b^
*Chemical scores (%)*			
Histidine	40.6 ± 1.1 ^b^	43.4 ± 0.7 ^a^	38.8 ± 1.0 ^b^
Isoleucine	55.4 ± 1.4 ^b^	58.7 ± 1.4 ^a^	39.9 ± 0.5 ^c^
Leucine	77.1 ± 2.9 ^b^	83.1 ± 3.4 ^a^	61.3 ± 1.5 ^c^
Lysine	23.8 ± 0.7 ^b^	28.6 ± 0.3 ^a^	21.4 ± 0.5 ^c^
Cysteine	55.7 ± 0.2 ^c^	63.6 ± 0.1 ^b^	73.7 ± 0.6 ^a^
Methionine	48.01 ±1.5 ^a^	51.5 ± 1.0 ^a^	51.0 ± 1.0 ^a^
Phenylalanine + Tyrosine	82.5 ± 2.8 ^b^	86.1 ± 3.1 ^a^	78.8 ± 1.8 ^c^
Threonine	27.2 ± 1.1 ^b^	31.5 ± 0.9 ^a^	33.3 ± 1.1 ^a^
Valine	41.3 ± 2.6 ^b^	44.8 ± 0.2 ^a^	45.8 ± 1.2 ^a^
Tryptophan	42.4 ± 3.1 ^b^	46.2 ± 0.7 ^a^	42.1 ± 0.2 ^b^
*Sequence of limiting EAA*			
	*Lysine*	*Lysine*	*Lysine*
	*Threonine*	*Threonine*	*Threonine*
	*Histidine*	*Histidine*	*Histidine*
Essential Amino Acid Index (EAAI)	45.7 ± 1.1 ^b^	50.3 ± 0.8 ^a^	45.2 ± 0.7 ^b^
Biological Value (BV)	38.1 ± 0.5 ^b^	43.1 ± 0.2 ^a^	37.5 ± 0.1 ^b^
Protein Efficiency Ratio (PER)	32.7 ± 0.4 ^a^	32.7 ± 0.7 ^a^	32.7 ± 0.5 ^a^
Nutritional Index (NI)	1.3 ± 0.1 ^b^	2.5 ± 0.1 ^a^	1.3± 0.1 ^b^
Hydrolysis Index (HI)	63.46 ± 1.99 ^b^	58.86 ± 1.25 ^c^	68.65 ± 1.47 ^a^
Predicted Glycemic Index (pGI)	74.55 ± 1.10 ^b^	72.02 ± 0.68 ^c^	77.35 ± 0.81 ^a^

The data are the means of three independent experiments ± standard deviations (*n* = 3). ^a–c^ Values in the same row with different superscript letters differ significantly (*p* < 0.05).

**Table 5:** Total phenolic compounds, expressed as mmol/kg of gallic acid (GA) equivalents, and antioxidant activity (radical scavenging activity on DPPH) of pasta containing fermented and native brewers’ spent grain: BSG-p, pasta containing native brewers’ spent grain (15% *wt/wt* in replacement of semolina); fBSG-p, pasta containing fermented brewers’ spent grain (15% *wt/wt* in replacement of semolina); W-p, pasta made with durum wheat semolina.

Pasta Sample	Status	Total Phenolic Compounds (mmol/kg GA eq)	Antioxidant Activity ^1^ (%)
**BSG-p**	Raw	0.69 ± 0.04 ^d^	33 ± 2 ^e^
	Cooked ***	0.70 ± 0.04 ^d^	26 ± 3 ^f^
	Digested ***	1.89 ± 0.08 ^b^	63 ± 2 ^b^
**fBSG-p**	Raw	0.77 ± 0.05 ^d^	40 ± 2 ^d^
	Cooked ***	0.85 ± 0.06 ^d^	35 ± 1 ^e^
	Digested ***	2.27 ± 0.06 ^a^	74 ± 3 ^a^
**W-p**	Raw	0.45 ± 0.04 ^e^	23 ± 2 ^f^
	Cooked ***	0.33 ± 0.02 ^e^	14 ± 2 ^g^
	Digested ***	1.46 ± 0.13 ^c^	57 ± 1 ^c^

^1^ The antioxidant activity was determined based on the scavenging activity towards DPPH radical after 10 min of reaction. The data are the means of three independent experiments ± standard deviations (*n* = 3). ^a–g^ Values in the same column with different superscript letters differ significantly (*p* < 0.05). *** Assays done on freeze-dried samples.

## Data Availability

The data used to support the findings of this study are available from the corresponding author upon request.

## Supplementary Materials

The following are available online at https://www.mdpi.com/article/10.3390/antiox10050742/s1, Table S1: Drying cycle used for pasta making. Table S2: List of the sensory attributes and their reference standards used for the sensory analysis of pasta.
