# Supplementary material for: Bioprocessed Brewers’ Spent Grain Improves Nutritional and Antioxidant Properties of Pasta
Source: Antioxidants (Basel). 2021 May 7;10(5):742. doi: 10.3390/antiox10050742 (PMC8151577; doi:10.3390/antiox10050742)
Supplement: Supplementary file 1 [file antioxidants-10-00742-s001.zip › antioxidants-1205551-supplementary.pdf]

Supplementary Table 1. Drying cycle used for pasta making.

	VENTILATION			RECOVERY		
**CYCLE**	Temperature (°C)	U.R.%	Time (min)	Temperature (°C)	U.R.%	Time (min)
**1**	55	85	3	55	85	2
**2**	55	85	3	55	85	2
**3**	55	70	3	55	70	4
**4**	55	70	3	40	65	8
**5**	55	70	3	40	60	10
**6**	55	70	3	40	60	10
**7**	55	70	3	40	55	10
**8**	55	70	3	40	55	12
**9**	55	70	3	40	55	12
**10**	55	70	3	40	55	12
**11**	55	70	3	40	55	12
**12**	55	70	3	40	55	14
**13**	55	70	3	40	50	14
**14**	55	70	3	40	50	16
**15**	55	70	3	40	50	16
**16**	55	70	3	40	50	16
**17**	55	65	3	40	50	16
**18**	55	65	3	40	50	18
**19**	55	65	3	40	50	18
**20**	55	65	3	40	45	18
**21**	55	65	3	40	45	18
**22**	55	60	3	35	45	18
**23**	55	60	3	35	45	18
**24**	50	55	3	35	45	18
**25**	45	55	3	35	15	18
**26**	40	40	3	15	15	10
**27**	15	10	20	15	10	20
**28**	15	10	20	15	10	20

Supplementary Table 2. List of the attributes and reference standards used for the sensory analysis of pasta.

Selected Attributes	Reference Standards
Appearance	
Evenness of colour<br>Homogeneity of texture (by looking)	Whole grain Pasta
Odor	
Cereal	Brewers' spent grain
Maltiness	Malt Extract (0.5%)
Sourdough Bread-like	Rye Bread
Overall intensity	
Texture	
Roughness	Extruded corn
Chewiness	Whole grain pasta
Taste/Flavor	
Whole grain	Whole grain bread
Bitterness	Caffeine solution (0.035%)
Overall Intensity	
